# Genomic diagnosis and multisystem phenotyping in pediatric congenital analbuminemia: clinical, coagulation, and immune signatures

**DOI:** 10.3389/fped.2026.1810945

**Published:** 2026-06-02

**Authors:** Asena Pinar Sefer, Melek Yorgun Altunbas, Baran Erman, Salim Can, Alper Bulutoglu, Satanay Hubrack, Katherine Ford, Melanie Makhlouf, Luis R. Saraiva, Gizem Onder, Ozden Hatirnaz, Ayse Merve Usta, Dilek Guller, Dilek Baser, Gamze Akgun, Umran Aba, Rahmi Kutay Erdogan, Omer Faruk Beser, Fugen Cullu Cokugras, Fatma Demirbas Ar, Nafiye Urganci, Oguz Salih Dincer, Sevgi Bilgic Eltan, Safa Baris, Elif Karakoc-Aydiner, Bernice Lo, Ahmet Ozen

**Affiliations:** 1 Division of Allergy and Immunology, Department of Pediatrics, School of Medicine, Recep Tayyip Erdogan University, Rize, Türkiye; 2 Division of Allergy and Immunology, Department of Pediatrics, School of Medicine, Marmara University, Istanbul, Türkiye; 3 The Isil Berat Barlan Center for Translational Medicine, Istanbul, Türkiye; 4 Institute of Child Health, Hacettepe University, Ankara, Türkiye; 5 Can Sucak Research Laboratory for Translational Immunology, Hacettepe University, Ankara, Türkiye; 6 Department of Translational Medicine, Sidra Medicine, Research Branch, Doha, Qatar; 7 College of Health & Life Sciences, Hamad Bin Khalifa University, Doha, Qatar; 8 Department of Comparative Medicine, Yale University School of Medicine, New Haven, CT, United States; 9 Department of Biochemistry and Molecular Biology, Health Science Institute, Acıbadem Mehmet Ali Aydınlar University, Istanbul, Türkiye; 10 Rare Diseases and Orphan Drugs Application and Research Center-ACURARE, Acıbadem University, Istanbul, Türkiye; 11 Division of Gastroenterology and Hepatology, Department of Pediatrics, Sarıyer Hamidiye Etfal Training and Research Hospital, Istanbul, Türkiye; 12 Division of Gastroenterology and Hepatology, Department of Pediatrics, Faculty of Medicine, Istanbul University Cerrahpasa, Istanbul, Türkiye; 13 Department of Pediatric Gastroenterology, Hepatology and Nutrition, Balıkesir Ataturk City Hospital, Ankara, Türkiye; 14 Division of Hematology and Oncology, Department of Pediatrics, School of Medicine, Recep Tayyip Erdogan University, Rize, Türkiye

**Keywords:** coagulation dysfunction, congenital analbuminaemia, hypoalbunimenia, immune dysfunction, multisystem involvement, protein-losing enteropathy, rare disease, whole-genome sequencing (WGS)

## Abstract

**Background:**

Congenital analbuminemia (CAA) is an ultrarare autosomal recessive disorder caused by biallelic pathogenic variants in the *ALB* gene, leading to absent or severely reduced serum albumin. Although historically considered a benign biochemical condition, emerging evidence suggests that pediatric CAA may be associated with clinically relevant multisystem manifestations.

**Methods:**

We conducted a longitudinal integrative study involving five children from two kindreds, all with genetically confirmed CAA. Their clinical features were thoroughly documented, along with extended coagulation tests and immunological profiling.

**Results:**

All patients presented with persistent hypoalbuminemia, early-onset edema, gastrointestinal morbidity (frequently leading to misdiagnosis as protein-losing enteropathy), antenatal complications, recurrent respiratory infections, and dyslipidemia. Genetic testing revealed an *ALB* splice-site mutation (c.1428 + 2T > C) in one kindred and a novel homozygous frameshift insertion (p.Glu69Ter) in the second. Coagulation profiling identified a reproducible prothrombotic signature, including elevated fibrinogen, D-dimer, and strikingly increased FXI activity, with one patient suffering life-threatening cerebral thrombosis. Immunological evaluation demonstrated preserved leukocyte and lymphocyte counts, preserved or elevated IgG levels, variable antigen-specific vaccine responses, reduced kappa-deleting recombination excision circle values in the tested patients, and skewing of CD4⁺ T cells toward effector memory subsets, consistent with immune dysregulation rather than frank immunodeficiency.

**Conclusions:**

This study provides detailed multisystem phenotyping of pediatric CAA and supports the view that CAA may present with clinically relevant gastrointestinal, metabolic, infectious, and hemostatic manifestations beyond isolated hypoalbuminemia. Early molecular diagnosis may reduce unnecessary invasive investigations and facilitate individualized monitoring for infectious and thrombotic complications. Larger multicenter studies with longitudinal follow-up and functional validation are required to confirm these findings.

## Introduction

Congenital analbuminemia (CAA; OMIM 616000) is a rare autosomal recessive (AR) disorder caused by biallelic pathogenic variants in the *ALB* gene, leading to absent or significantly reduced serum albumin levels (1, 2). Fewer than 100 cases have been reported worldwide, with an estimated prevalence of less than 1 in 1,000,000 live births. However, the actual frequency is likely underestimated because conventional albumin assays tend to overestimate albumin levels at very low concentrations, and many affected individuals remain asymptomatic or present only with non-specific symptoms (2–4). Severe hypoalbuminemia in CAA often mimics other causes of hypoalbuminemia, such as protein-losing enteropathy (PLE) or nephrotic syndrome, leading to delayed or missed diagnosis (1). Therefore, molecular confirmation is essential for a definitive diagnosis (2).

Clinically, CAA is characterized by profound hypoalbuminemia, leading to a relative increase in non-albumin plasma proteins and metabolic disturbances, including dyslipidemia (5). The phenotype varies with age: adults frequently present with a milder course due to compensatory mechanisms, whereas pediatric patients present with generalized edema, growth disturbances, dyslipidemia, and increased perinatal morbidity and mortality (2, 4, 6, 7).

Emerging evidence suggests that CAA has important systemic consequences rather than representing a benign biochemical state. Profound hypoalbuminemia can disrupt hemostatic balance and endothelial integrity, and dyslipidemia contributes to vascular risk (8, 9). Venous thrombosis and even early myocardial infarction have been documented in young patients, raising concerns about a predisposition to vascular disease from early life (9–12). Moreover, recurrent and severe respiratory tract infections (RTIs) are increasingly recognized as part of the pediatric phenotype, in keeping with the critical role of albumin in transport functions, antioxidant defense, and immunomodulation (1, 2, 4, 7, 13).

Despite these observations, the pathophysiological consequences of CAA remain incompletely understood. In particular, the thromboembolic (TE) risk profile and detailed immunological features of pediatric CAA have not been systematically investigated.

In this study, we report five pediatric patients from two kindreds who were initially misdiagnosed with PLE and subsequently confirmed to have CAA by trio whole-genome sequencing (WGS). By combining longitudinal clinical evaluation with comprehensive coagulation and immunological analyses, we aimed to refine the characterization of pediatric CAA and to provide insights into its relevance as a model condition at the intersection of albumin deficiency, vascular risk, and immune homeostasis.

## Methods

### Study design, study population, and ethical approval

We conducted a mixed retrospective–prospective, longitudinal observational cohort study from June 2019 to September 2024. Long-term retrospective clinical data were combined with a prospective systematic evaluation of coagulation and immunological parameters.

The cohort comprised five children with persistent unexplained hypoalbuminemia who had been under long-term follow-up with a presumptive diagnosis of PLE. Comprehensive evaluations, including urinalysis, urine protein-to-creatinine ratio, and liver function testing, excluded renal protein loss and hepatic synthetic dysfunction. As part of the study protocol, trio WGS revealed biallelic pathogenic variants in the *ALB* gene in all the patients, which were validated by Sanger sequencing, confirming the diagnosis of CAA.

Age-matched control groups were selected according to the objective of each analysis. Patients with clinically confirmed PLE, defined as persistent hypoalbuminemia with evidence of gastrointestinal (GI) protein loss, were used as the main control group for both coagulation and immunological assessments. PLE was selected because it shares the key biochemical feature of hypoalbuminemia with CAA, but results from secondary intestinal albumin loss rather than congenital absence or severe reduction of albumin synthesis. This comparison, therefore, provided a clinically relevant framework for interpreting whether findings observed in CAA were also present in secondary hypoalbuminemia or were more specific to congenital albumin deficiency. For immunological analyses, additional age-matched control groups with well-defined inborn errors of immunity (IEI) were included. CHAPLE disease was selected because it combines PLE, hypoalbuminemia, and monogenic IEI. Ataxia-telangiectasia (AT) was included as a well-characterized cellular immunodeficiency without hypoalbuminemia. Age-matched healthy pediatric controls were included for selected immunological comparisons.

The study was approved by the Ethics Committees of Marmara University and Sidra Medicine and performed in accordance with the Declaration of Helsinki. Written informed consent was obtained from parents or legal guardians, and assent was obtained from children when appropriate. The same consent and assent procedures were applied to healthy controls and all disease comparator groups. Additional written informed consent was obtained from the parent for the publication of identifiable clinical photographs.

### Whole-genome sequencing and variant analysis

Genomic DNA was extracted from peripheral blood samples using the QIAamp DNA Blood Mini Kit (Qiagen, The Netherlands). Trio WGS was performed at Sidra Medicine (Qatar) using the Illumina HiSeq X platform with an average coverage of 30×. Sequencing reads were filtered for quality and aligned to the human reference genome (GRCh37/hg19) using the Burrows–Wheeler Aligner with default parameters. Variant calling and annotation were performed with the Genome Analysis Toolkit (GATK) by the Sidra Bioinformatics Core. Variant interpretation followed standard rarity- and inheritance-based prioritization and was classified according to the American College of Medical Genetics and Genomics and the Association for Molecular Pathology (ACMG/AMP) guidelines for sequence variant interpretation. The evidence categories considered included population frequency data, predicted functional consequence (e.g., loss-of-function and splice-site impact), segregation with disease within families, phenotype consistency, and computational predictions used as supportive evidence. *In silico* prediction tools (including SIFT, PolyPhen-2, and CADD) were used as supporting evidence. An in-house composite ranking metric (Variant Analysis with Multiple Pathogenicity Predictors; VAMPP) was used only as an auxiliary prioritization tool; its implementation and weighting scheme are provided in the Supplementary Materials. Candidate variants were confirmed by Sanger sequencing.

### Clinical and laboratory evaluations

Patients underwent a structured clinical assessment, including demographic data, age at presentation, GI manifestations, growth parameters, and treatment history. Retrospective clinical variables were abstracted from medical records, while prospective follow-up captured interval clinical events and, when available, contemporaneous laboratory findings.

TEs were defined as clinically suspected episodes confirmed by imaging and/or specialist documentation. The timing, clinical context, treatment, outcome, and available provoking risk factors were recorded. Infectious history was assessed retrospectively from medical records and prospectively during follow-up. Recurrent infections were defined according to established criteria used in the evaluation of IEI, including ≥2 episodes of pneumonia per year, ≥4 episodes of otitis media per year, ≥2 episodes of sinusitis per year, or ≥2 episodes of sepsis (14, 15). Severe infection was defined as an infection requiring hospitalization, intravenous antimicrobial treatment, or intensive care support. Microbiological documentation, including sputum and blood culture results, was recorded when available. Episodes without microbiological confirmation were classified according to physician-documented diagnosis, radiological findings, treatment requirement, and hospitalization records.

Laboratory investigations included a complete blood count, liver and kidney function tests, lipid profile, and quantitative serum protein measurements. Protein electrophoresis was carried out to confirm the absence or significant reduction of albumin and to assess compensatory increases in non-albumin proteins.

For immunological and coagulation analyses, prospective blood samples from patients with CAA and control groups were preferably obtained under predefined clinical conditions, during clinically stable, infection-free periods, and outside periods of fever, hospitalization, or recent clinical deterioration. The timing of blood sampling relative to the albumin infusion was carefully considered. When feasible, samples were collected at least 5–7 days after the most recent albumin infusion. This pragmatic sampling window was chosen to minimize immediate postinfusion changes in intravascular volume, serum albumin concentration, and albumin-dependent immune or coagulation parameters, given the known distribution kinetics and variable volume effects of infused albumin (16).

### Coagulation and thrombotic risk assessment

Extended coagulation testing was performed in all patients, including prothrombin time (PT), activated partial thromboplastin time (aPTT), fibrinogen, D-dimer, antithrombin III activity, protein C and protein S activity, and coagulation factor (F) levels (FII, FV, FVII, FVIII, FIX, FX, and FXI), measured using clot-based or chromogenic assays with institutional laboratory protocols. Age-appropriate reference ranges were applied when available. Patients with CAA (*n* = 5) were compared with age-matched clinically stable patients with PLE with available coagulation data (*n* = 13).

### Immunological evaluation

Serum immunoglobulin (Ig) levels (IgG, IgA, IgM, and total IgE) were measured alongside vaccine-induced antibody responses, including anti-mumps IgG, anti-HBs, anti-rubeola IgG, and anti-pneumococcus IgG. Lymphocyte subpopulation analysis was performed to assess T and B cells and their subsets, as previously described (17), and the results were compared with age-specific normative data (17). T-cell receptor excision circles (TRECs) and kappa-deleting recombination excision circles (KRECs) were quantified using quantitative real-time polymerase chain reaction (qPCR). Copy numbers were determined and interpreted in relation to age-matched reference values. T-cell receptor (TCR) immune profiling was carried out using established methods (18) to evaluate T-cell diversity and clonality. Patients with CAA (*n* = 5) were compared with age-matched disease comparator groups, including PLE (*n* = 42), CHAPLE disease (*n* = 38), and AT (*n* = 15). For TCR repertoire analysis, one patient with CAA was compared with three age-matched healthy controls.

All analyses were conducted in accordance with institutional protocols. The full antibody panels (including clones/fluorochromes), gating strategy, and cytometer quality control procedures, details of TCR A-B library preparation, sequencing, bioinformatic processing, diversity metric definitions, and normalization procedures are provided in the Supplementary Materials.

### Statistical analysis

Numerical variables are expressed as either mean ± standard deviation (SD) or median with interquartile ranges (IQR; 25%–75%), depending on the distribution assessed by the Shapiro–Wilk test. Given the small sample size, analyses were primarily descriptive. Non-parametric tests were used for group comparisons where appropriate, including the Mann–Whitney *U*test for two-group comparisons and the Kruskal–Wallis test with Dunn's *post hoc* correction for multiple-group comparisons. A *p*-value <0.05 was considered statistically significant. Because of the limited sample size, cross-sectional sampling of immune and coagulation parameters, and heterogeneity in treatment exposure, *p*-values were interpreted descriptively rather than as definitive evidence of disease-specific effects. Statistical analyses and graph creation were performed using GraphPad Prism version 10.6.0 (GraphPad Software Inc., San Diego, CA, USA) and Adobe Illustrator 25.2.1 (Adobe Inc., CA, USA).

## Results

### Demographic and clinical features

**Figure 1:**
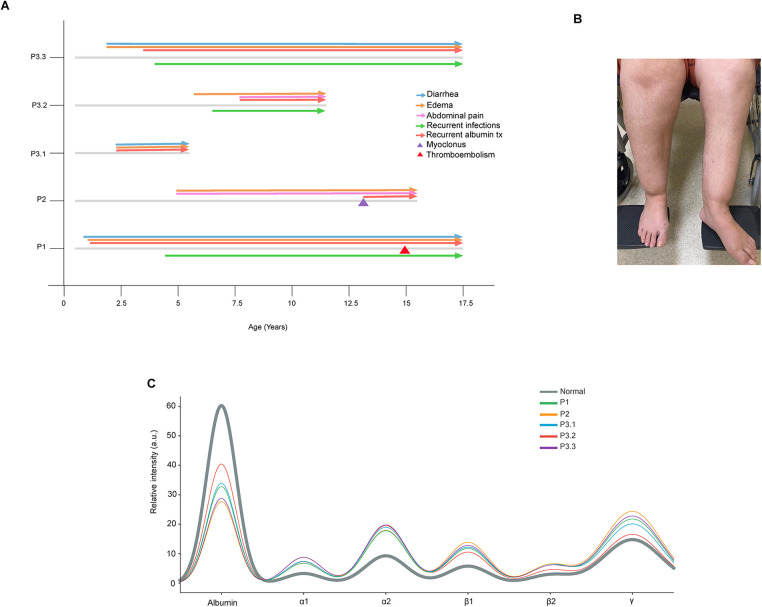
Clinical course, representative clinical features, and serum protein electrophoresis in patients with congenital analbuminemia (CAA). **(A)** Timeline of major clinical manifestations and treatment requirements in affected individuals. Colored arrows indicate the period during which a given manifestation or the need for therapy began and continued thereafter. Triangles denote discrete events. The horizontal axis indicates patient age (years). **(B)** Representative photograph showing lipodystrophy in P1. **(C)** Serum protein electrophoresis profiles showing markedly reduced albumin fractions in patients compared with a reference control, with relative increases in non-albumin fractions. *α*1, *α*2, *β*1, *β*2, *γ*: globulin fractions, a.u., arbitrary units; tx, treatment.

Five Five female patients with persistent hypoalbuminemia were identified from two unrelated kindreds. Kindred 1 included Patient (P) 1, whereas Kindred 2 included P2 and siblings P3.1, P3.2, and P3.3, who were related through extended consanguinity. The median age at disease onset was 24 months (IQR, 12–54), and the median age at last follow-up was 15 years (IQR, 11–17) (Table 1). Antenatal complications were documented in 2/5 patients (40%), including polyhydramnios in P2 and hydrops fetalis in P3.3. The predominant presenting manifestations were edema, mainly periorbital or lower-extremity edema, in all patients, watery diarrhea in 3/5 patients (60%), and abdominal pain in 2/5 patients (40%) (Figure 1A). P2 presented atypically with unilateral hand myoclonus due to severe hypocalcemia. Lower-extremity lipodystrophy developed after the age of 15 years in 2/5 patients (40%; P1 and P3.3) (Figure 1B). No dysmorphic features were observed, and growth parameters were relatively preserved (Table 1).

**Table 1:** Demographic and key clinical characteristics of pediatric patients with congenital analbuminemia (OMIM: 616000).

Patient	Last documented age (y)/ sex assigned at birth	Age at onset (mo)	Antenatal history	Presenting symptoms	Recurrent infections	Severe infections	Weight/height (SDS)	TE event	Endoscopy-pathology
P1	17/ F	2	None	Diarrhea, edema	LRTI, URTI	Sepsis	−1.5/ −0.59	Basilar artery thrombosis	IL
P2	15/ F	54	Polyhydramnios	Myoclonus, abdominal pain, edema	None	None	−0.98/ −0.92	-	Normal
P3.1	5/ F	24	None	Edema, diarrhea	None	None	1.21/ 0.5	-	Ectatic lymphatic vessels
P3.2	11/ F	60	None	Abdominal pain, edema	URTI	None	−0.8/ −1.0	-	Normal
P3.3	17/ F	12	Hydrops Fetalis	Edema, diarrhea	LRTI, URTI	Pneumonia	−1.8/ −1.4	-	Lymphoplasmacytic cell infiltration in the lamina propria

F, female; IL, intestinal lymphangiectasia; LRTI, lower respiratory tract infection; mo, month; SDS, standard deviation score; TE, thromboembolic; URTI, upper respiratory tract infection; y, year.

All patients experienced GI symptoms from early childhood, consistently accompanied by persistent hypoalbuminemia, and were followed long-term by pediatric gastroenterology under a presumptive diagnosis of PLE. Initial endoscopic and histopathological assessments showed intestinal lymphangiectasia in P1; however, this finding was not confirmed in subsequent examinations. Ectatic lymphatic vessels were identified in P3.1, and lymphoplasmacytic infiltration of the lamina propria was observed in P3.3, while non-specific mucosal changes were reported in the remaining patients (Table 1).

All patients required long-term supportive treatment during follow-up. Intravenous albumin replacement, at a standard dosage of 1 gr/kg/ per infusion, was the main therapeutic intervention and was guided primarily by clinical deterioration rather than serum albumin concentration alone. During clinically stable periods, albumin was generally administered once or twice monthly. However, the requirement increased during episodes of clinical worsening, particularly acute infections. During some inpatient episodes, albumin infusions were required as frequently as every other day. Historical data on per-infusion dose, cumulative exposure, and exact infusion timing were not reliably available across the entire follow-up period due to the long-term follow-up period before study enrollment.

Supportive treatments commonly used for suspected PLE, including a high-protein diet and medium-chain triglyceride (MCT) supplementation, were prescribed in all patients. MCT supplementation was administered for a mean duration of 7.0 ± 2.5 months. Based on available clinical records, this intervention was not associated with a clear improvement in symptoms or reduction in albumin replacement requirement. P1 also received a 3-month course of somatostatin for presumed PLE, without clear clinical benefit. At the last follow-up, all patients were alive; however, edema remained recurrent, and GI symptoms, particularly abdominal pain with intermittent diarrhea, persisted episodically (Figure 1A).

### Genetic findings

**Figure 2:**
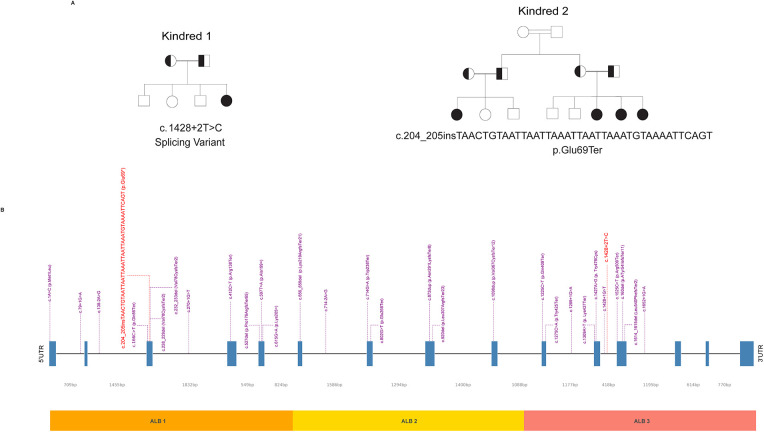
Pedigrees and schematic representation of the *ALB* gene with protein domains and the distribution of pathogenic variants. **(A)** The pedigrees of the two consanguineous kindreds illustrate the autosomal recessive inheritance of the *ALB* variants. Squares represent male individuals, circles represent female individuals, and double horizontal lines indicate parental consanguinity. Genotypes are shown below each pedigree. Half-filled symbols represent heterozygous carriers, filled symbols indicate homozygous individuals, and empty symbols denote either wild-type (WT/WT) or non-genotyped individuals. **(B)** Schematic representation of the *ALB* gene and protein structure with previously reported pathogenic variants (purple) and variants identified in this study (red). The albumin protein comprises three homologous domains (I–III) with multiple binding sites for fatty acids, bilirubin, and drugs; reported pathogenic variants are distributed throughout the coding sequence. Alb, albumin; UTR, untranslated region; bp, base pair.

Trio WGS identified two disease-attributable variants in *ALB* across the cohort (Table 2; Figures 2A,B). P1 harbored a canonical splice-site variant (NM_000477.7: c.1428 + 2T > C), previously linked to CAA (19), which disrupts the splice donor site and was classified as pathogenic according to the ACMG/AMP guidelines (PVS1, PM2). Supporting evidence included a high Combined Annotation-Dependent Depletion (CADD) score (25.3) and strong evolutionary conservation indicated by the Genomic Evolutionary Rate Profiling (GERP++) score of 5.5.

**Table 2:** Genetic findings in patients with congenital analbuminemia, including homozygous *ALB* variants (NM_000477.7, chromosome 4) with detailed variant annotation and pathogenicity assessment.

Patients	Position	Sequence ontology	cDNA change	Protein change	rsID	ClinVar ID	CADD exome PHRED	VAMPP ranked score	GERP++	GnomAD allele frequency	ACMG criteria
P1	74283388	Non-Coding	c.1428 + 2T > C	Non-Coding	rs78784172	156311	25.3	NA	5.5	NA	Pathogenic (PVS1, PM2)
P2, P3.1, P3.2, P3.3	74272411	Nonsense	c.204_205insTAACTGTAATTAATTAAATTAATTAAATGTAAAATTCAGT	p.Glu69*	NA	NA	NA	NA	NA	NA	Pathogenic (PVS1, PM2)

ACMG, American College of Medical Genetics and Genomics; ALB, albumin gen; CADD, Combined Annotation Dependent Depletion; ClinVar, clinical variants database; GERP++, Genomic Evolutionary Rate Profiling; GnomAD, Genome Aggregation Database; Hom, homozygous; NA, not available; NM, reference transcript accession number (RefSeq); PVS1, ACMG criterion for “pathogenic very strong” (null variant in a gene where loss of function is a known mechanism of disease); PM2, ACMG criterion for “pathogenic moderate” (absent/rare in population databases); rsID, reference SNP cluster identification; VAMPP, Variant Annotation, Mapping, and Prediction Platform.

In P2, 3.1, 3.2, and 3.3, a novel homozygous insertion in *ALB* (NM_000477.7: c.204_205insTAACTGTAATTAATTAAATTAATTAAATGTAAAATTCAGT) was identified. This frameshift variant is predicted to introduce a premature termination codon (p.Glu69Ter) and represents a loss-of-function allele. This predicted null allele, not previously described and absent from ClinVar, is also absent from the Genome Aggregation Database (gnomAD), indicating it is a novel pathogenic mutation. According to ACMG/AMP criteria, it was classified as pathogenic (PVS1, PM2) based on its predicted loss-of-function effect, absence from population databases, and segregation with the disease in the affected family.

Segregation analysis confirmed biallelic AR inheritance: all affected individuals were homozygous for these variants, while both parents were heterozygous carriers (Figure 2A). All reported *ALB* variants were confirmed by Sanger sequencing. No additional pathogenic/likely pathogenic variants consistent with the clinical phenotype were identified on trio WGS.

### Laboratory findings

All patients had persistent hypoalbuminemia without evidence of renal protein loss or hepatic failure. The mean serum albumin level was 2.21 ± 0.64 g/dL and the total protein was 5.49 ± 0.73 g/dL. Dyslipidemia was present in all patients, with elevated LDL cholesterol (mean 151.8 ± 13.2 mg/dL), hypertriglyceridemia (mean 176.6 ± 17.4 mg/dL), and variable HDL cholesterol levels (mean 51.2 ± 12.7 mg/dL), including a significantly reduced level in one patient (29 mg/dL) (Table 3). Serum protein electrophoresis revealed a decrease in albumin fraction, with compensatory increases in α2- and β-globulin fractions (Figure 1C).

**Table 3:** Hematological, biochemical, and coagulation parameters in patients with congenital analbuminemia.

Parameter (reference)	P1	P2	P3.1	P3.2	P3.3	Mean (±SD)
CBC						
Hemoglobin (12–16 g/dL)	**10** **.** **8**	**11** **.** **5**	12	**11** **.** **2**	**10** **.** **5**	11.2 ± 0.59
Platelet count (150–450 × 10⁹/L)	420	395	380	360	410	393.0 ± 23.9
Protein profile						
Serum albumin (3.5–5.5 g/dL)	**1** **.** **6**	**1** **.** **8**	**2** **.** **9**	**1** **.** **84**	**2** **.** **9**	2.21 ± 0.64
Total protein (6–8 g/dL)	**4** **.** **5**	**5** **.** **2**	**5** **.** **4**	**5** **.** **97**	**6** **.** **4**	5.49 ± 0.73
Lipid profile						
LDL (<110 mg/dL)	**160**	**145**	**132**	**158**	**164**	151.8 ± 13.2
HDL (>54 mg/dL)	60	**53**	**29**	58	56	52.2 ± 12.7
Triglycerides (<150 mg/dL)	**180**	**167**	**158**	**204**	**174**	176.6 ± 17.4
Coagulation						
PT (11–15 s)	12.4	13.8	12.4	13.5	13.6	13.1 ± 0.57
aPTT (26–34 s)	**36**	**25** **.** **9**	29.3	28.8	28.2	29.6 ± 3.8
Fibrinogen (200–400 mg/dL)	**436**	**462**	**607**	**608**	**590**	540.6 ± 84.4
D-dimer (0.00–0.05 mg/dL)	**1** **.** **4**	**0** **.** **8**	**0** **.** **6**	**0** **.** **5**	**0** **.** **5**	0.76 ± 0.38
AT-III (80%–120%)	111	118	**133**	**135**	**133**	126.0 ± 10.8
F-V (60%–150%)	**56**	**55**	143	**186**	**175**	123.0 ± 63.6
F-IX (60%–150%)	116	116	133	**163**	**165**	138.6 ± 24.2
F-X (60%–150%)	106	88	**155**	**164**	**153**	133.2 ± 33.9
F-XI (60%–150%)	118	**300**	**200**	**200**	**200**	203.6 ± 64.5
Pr-C (60%–150%)	**174**	142	130	**183**	**200**	165.8 ± 29.1
Pr-S (60%–150%)	105	111	**175**	135	**194**	144.0 ± 39.2

aPTT, activated partial thromboplastin time; AT-III, antithrombin III; CBC, complete blood count; F, factor; HDL, high-density lipoprotein; LDL, low-density lipoprotein; P, patient; Pr-C, Protein C; Pr-S, Protein S; PT, prothrombin time; SD, standard deviation; sec, second.

Values are shown for individual patients and as mean ± standard deviation. Reference ranges are indicated in parentheses.

Values outside the reference range are shown in bold.

### Thromboembolic complications and coagulation analyses

**Figure 3:**
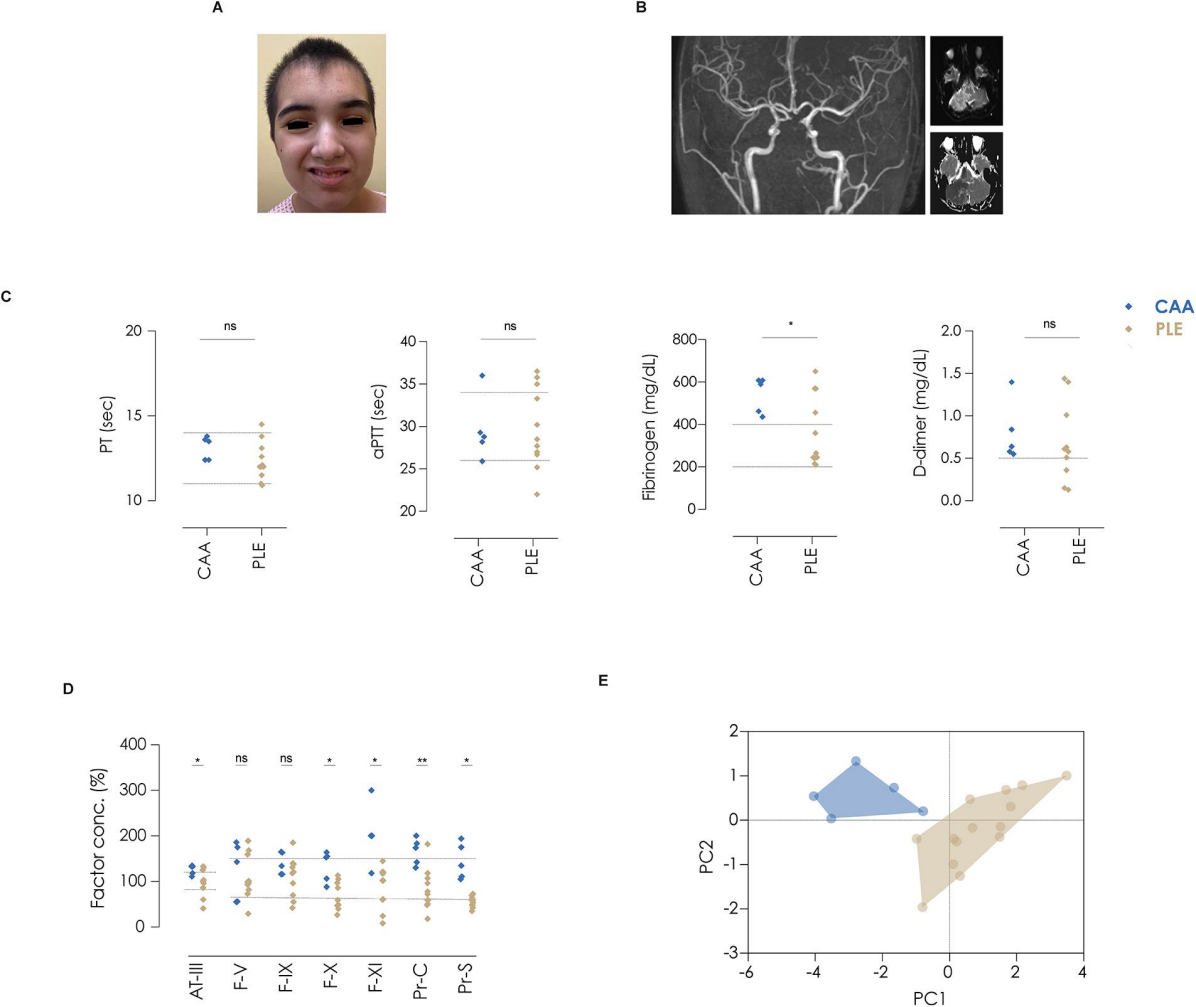
Thromboembolic complications and blood coagulation marker abnormalities in patients with congenital analbuminemia (CAA). **(A)** Representative image of P1 showing facial paralysis following a cerebrovascular thromboembolic event. **(B)** Magnetic resonance imaging (MRI) angiography of P1 showing basilar artery occlusion (left, larger image). Diffusion-weighted MRI and apparent diffusion coefficient map images reveal an acute-subacute infarct in the right cerebellar hemisphere and pons. **(C)** Comparison of PT, aPTT, fibrinogen, D-dimer, and **(D)** serum coagulation factor concentrations between patients with CAA and disease controls with protein-losing enteropathy (PLE). Each symbol represents an individual patient, color-coded by disease group**.** Horizontal dotted lines indicate the upper and lower limits of the normal range. **(E)** Exploratory principal component analysis based on the coagulation parameters shown in C and D. Group comparisons were performed with Mann–Whitney *U* tests for each analyte. A significance threshold of *p* < 0.05 was used. *p < 0.05, **p < 0.005, ***p < 0.001. aPTT, activated partial thromboplastin time; CAA, congenital analbuminemia; AT-III, antithrombin III; F-V, factor V; F-IX, factor IX; F-X, factor X; F-XI, factor XI; PC1, principal component 1; PC2, principal component 2; Pr-C, protein C; Pr-S, protein S; PLE, protein-losing enteropathy; PT, prothrombin time; sec, second.

Among the five patients, one patient (P1) experienced a TE event. At 15 years of age, she developed acute neurological symptoms, including facial paralysis, and cranial imaging demonstrated intracranial thrombosis with multiple cerebral infarctions (Figures 3A,B). She required intensive care, urgent thrombectomy, and anticoagulation therapy. The clinical course was complicated by cerebral edema requiring external ventricular drainage and decompressive craniectomy. At the time of the TE event, no acquired provoking risk factors were identified, including central venous catheterization, immobilization, oral contraceptive use, smoking, obesity, acute infection, dehydration, recent surgery, or trauma. Thrombophilia screening, including antiphospholipid antibodies, factor V Leiden, prothrombin G20210A mutation, and plasma homocysteine, was negative. She had major neurological sequelae and remained wheelchair-dependent, requiring ongoing rehabilitation, anticoagulation, and regular albumin replacement.

Extended coagulation testing was performed in all patients (Table 3). PT and aPTT values were within or close to reference ranges on average, with mean PT of 13.1 ± 0.7 and mean aPTT of 29.6 ± 3.8 s. In contrast, fibrinogen levels were elevated in all patients (mean 540.6 ± 84.4 mg/dL), and D-dimer levels were increased (mean 0.76 ± 0.38 mg/dL). Coagulation factor activities showed a heterogeneous pattern. FV activity was reduced in P1 and P2 but increased in P3.1–P3.3, whereas FIX and FX activities were largely preserved or increased. FXI activity was elevated in 4/5 patients (80%), reaching up to 300%. Natural anticoagulant activities were also frequently increased, including protein C and protein S.

To determine whether the observed coagulation profile could be attributed solely to marked hypoalbuminemia, coagulation parameters were compared with those from a disease-control group of children with PLE. In these exploratory comparisons, fibrinogen levels were higher in CAA than in PLE (*p* = 0.04), whereas PT, aPTT, and D-dimer did not differ significantly between groups (*p* = 0.12, *p* = 0.9, and *p* = 0.4, respectively) (Figure 3C). Among individual coagulation factors and natural anticoagulants, AT-III activity was higher in CAA than in PLE (*p* = 0.03), while FV and FIX activities were comparable between groups (*p* = 0.58 and *p* = 0.12, respectively). FX, FXI, protein C, and protein S activities were also higher in CAA than in PLE (*p* = 0.017, *p* = 0.018, *p* = 0.004, and *p* = 0.017, respectively) (Figure 3D). Exploratory principal component analysis (PCA) integrating fibrinogen, D-dimer, PT, aPTT, coagulation factor activities, and natural anticoagulant parameters showed separation between CAA and PLE samples along the first two principal components (Figure 3E).

### Infectious complications and immunological analyses

Recurrent infections were documented in 3/5 patients (60%) according to predefined criteria and predominantly involved the respiratory tract, with onset in early childhood (Figure 1A). Severe infections requiring hospitalization and/or intravenous antimicrobial treatment occurred in 2/5 patients (40%). P1 had recurrent lower RTI, including severe pneumonia complicated by sepsis; during one episode, *Haemophilus influenzae* was isolated from sputum and *Staphylococcus aureus* from blood culture. P3.3 had recurrent pneumonia, with sputum cultures yielding *Streptococcus pneumoniae*. P3.2 had recurrent upper RTI, although no invasive bacterial infection or culture-confirmed severe infection was documented (Table 1).

Baseline immunological evaluation showed preserved leukocyte, neutrophil, and lymphocyte counts in all patients (Supplementary Table S1). Serum Ig profiling revealed elevated IgG levels in 2/5 patients (40%), whereas IgA and IgM levels were within age-adjusted reference ranges in all patients. IgE levels were increased in all patients, without clinical evidence of allergic disease or parasitic infection. Vaccine responses to hepatitis B and rubella were preserved in all patients; however, absent responses to mumps and pneumococcal antigens were observed in P1 and P2 (Table 4).

**Table 4:** Key immunological findings in patients with congenital analbuminemia.

Subject	P1	P2	P3.1	P3.2	P3.3
Immunoglobulins					
IgA (mg/dL)	289 (108–477)	230 (96–465)	78 (71–235)	181 (69–387)	199 (108–477)
IgG (mg/dL)	**2,400** **(↑)** (876–2,197)	**1,980** **(↑)** (987–1,958)	869 (640–2,810)	1,276 (764–2,134)	1,475 (876–2,197)
IgM (mg/dL)	310 (75–448)	280 (83–282)	183 (44–244)	156 (78–383)	174 (75–448)
IgE (IU/mL)	**250 (↑)**	**126 (↑)**	**348 (↑)**	**100 (↑)**	**89 (↑)**
Vaccine responses					
Anti-Hbs	Positive	Positive	Positive	Positive	Positive
Anti-mumps	Negative	Negative	Positive	Positive	Positive
Anti-rubeola	Positive	Positive	Positive	Positive	Positive
Anti-pneumococcus	Negative	Negative	Positive	Positive	Positive
TREC^a^	998	1,150	NA	NA	NA
KREC^b^	**1,320 (↓)**	**914 (↓)**	NA	NA	NA
Lymphocyte subsets					
CD3+ T cells (%)	63 (59–88)	77 (59–88)	71 (58–83)	75 (56–89)	82 (59–88)
CD3 + CD4+ T cells (%)	39 (28–48)	46 (28–48)	41 (25–55)	46 (25–51)	45 (28–48)
CD3 + CD8+ T cells (%)	18 (18–43)	20 (18–43)	24 (14–39)	26 (18–43)	23 (8–43)
CD19+ B cells (%)	**26** **(↑)** (5–21)	14 (5–21)	21 (10–31)	17 (7–23)	12 (5–21)
CD16 + 56+ NK cells (%)	10 (5–35)	6 (5–35)	5 (3–30)	6 (4–29)	6 (5–35)
Naive B cells (%)	65 (46–92)	70 (46–92)	88 (55–95)	77 (55–90)	77 (46–92)
UCSM B cells (%)	11 (5–28)	26 (5–28)	**3** **(↓)** (6–23)	18 (6–28)	9 (5–28)
CSM B cells (%)	12 (6–35)	**2** **(↓)** (6–35)	**1** **(↓)** (3–32)	**3** **(↓)** (7–31)	**3** **(↓)** (6–35)
RTE (%)	**22** **(↓)** (28–61)	37 (28–61)	59 (49–78)	**34** **(↓)** (37–73)	**27** **(↓)** (28–61)
Naive CD4+ T cells (%)	32 (31–70)	42 (31–70)	65 (49–90)	47 (40–79)	42 (31–70)
CM CD4+ T cells (%)	**10** **(↓)** (26–53)	17 (26–53)	**9** **(↓)** (13–44)	**14** **(↓)** (17–53)	**12** **(↓)** (26–53)
EM CD4+ T cells (%)	**56** **(↑)** (4–23)	**35** **(↑)** (4–23)	**13** **(↑)** (1–10)	**36** **(↑)** (2–14)	23 (4–23)

anti-HBs, hepatitis B surface antibody; CD, cluster differentiated; CM, central memory; CSM, class-switched memory; EM, effector memory; Ig, immunoglobulin; KREC, kappa-deleting recombination excision circle; NA, not available; NK, natural killer; RTE, recent thymic emigrants; TREC, T-cell receptor excision circle; UCSM, unclass-switched memory.

Values are shown for individual patients. Age-adjusted reference ranges are indicated in parentheses where applicable. ↑: Above age-matched reference range, ↓: Below age-matched reference range. Values outside the age-specific reference ranges are indicated in bold. TREC and KREC values are expressed as copies/10⁶ cells.

a TREC values are expressed as copies/10⁶ cells. The reference value used was median 12,600 copies/10⁶ cells, with a reference range of 720–36,200.

b KREC values are expressed as copies/10⁶ cells. The reference value used was median 11,335 copies/10⁶ cells, with a reference range of 1,720–61,000.

Lymphocyte subset analysis demonstrated preserved absolute counts and overall distribution of total T cells, CD4⁺ T cells, CD8⁺ T cells, B cells, and natural killer (NK) cells. CD8⁺ T-cell subsets did not show a consistent abnormal pattern across the cohort. In contrast, selected abnormalities were observed within the CD4⁺ T-cell memory compartment. Naive CD4⁺ T cells were largely preserved, whereas central memory (CM) CD4⁺ T cells were reduced in the majority of the patients, and effector memory (EM) CD4⁺ T cells were increased in 4/5 patients (80%). Recent thymic emigrants (RTE) were close to the lower reference range or mildly reduced in 3/5 patients (60%). However, TREC values, available in 2/5 patients, were within the expected range in both tested patients. In contrast, KREC values were reduced in both tested patients (2/2), and class-switched memory (CSM) B cells were reduced in 4/5 patients (80%). Key immunological findings are summarized in Table 4, and extended immunophenotyping results are provided in Supplementary Table S1.

**Figure 4:**
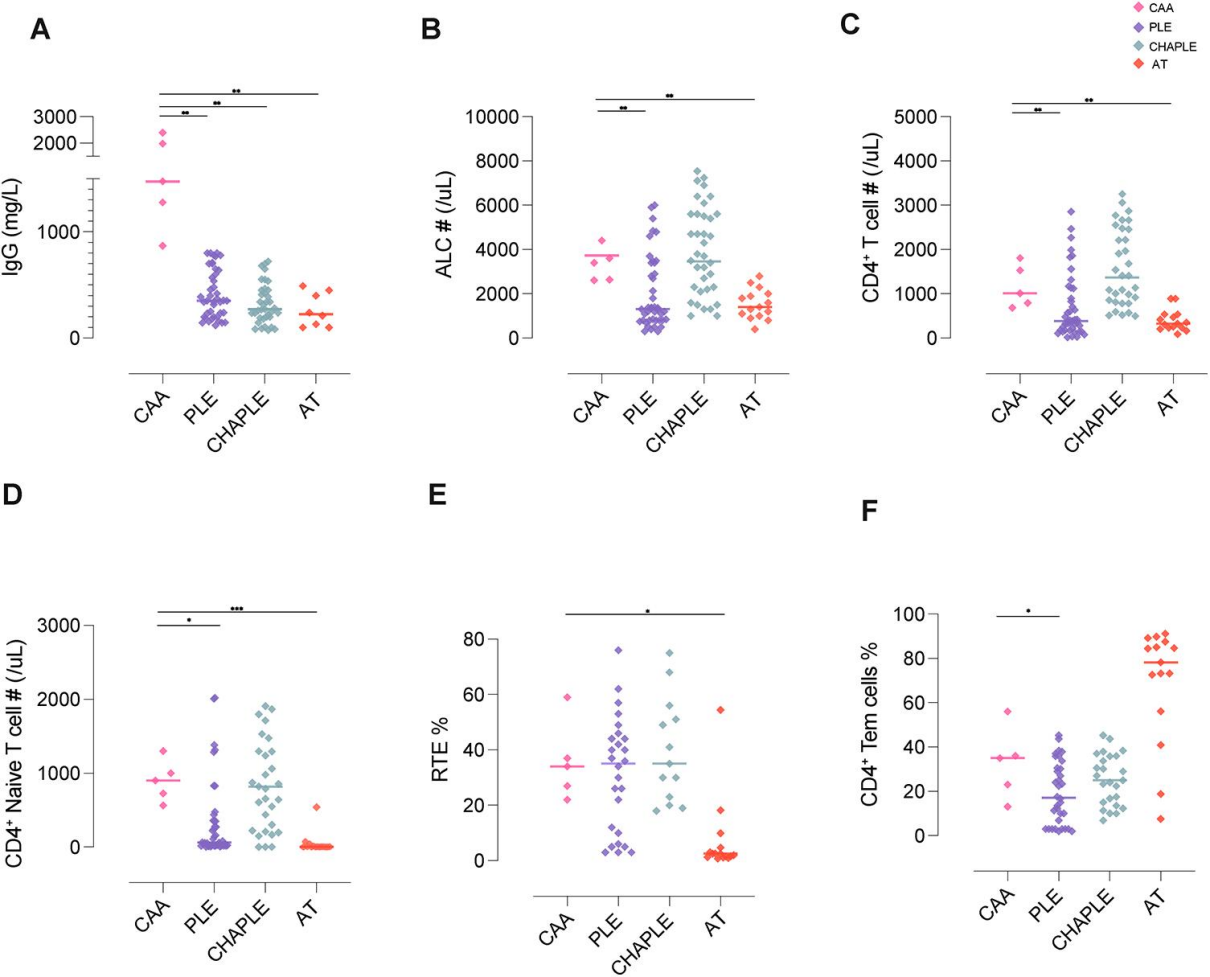
Immunological characteristics of patients with congenital analbuminemia compared with those with protein-losing enteropathy, CHAPLE disease, and ataxia-telangiectasia. **(A)** Serum IgG levels (mg/L). **(B)** Absolute lymphocyte counts (ALC,/µL). **(C)** Total CD4⁺ T-cell counts (/µL). **(D)** CD4⁺ naive T-cell counts (/µL). **(E)** Percentage of recent thymic emigrant cells (RTE, %). **(F)** Percentage of CD4⁺ effector memory cells. Each symbol represents an individual patient, with colors distinguishing disease groups. Horizontal lines indicate median values. Statistical analysis was performed using the Kruskal–Wallis test followed by Dunn's multiple-comparison *post hoc* test. *p < 0.05, **p < 0.005, ***p < 0.001. ALC, absolute lymphocyte count; AT, ataxia-telangiectasia; CAA, congenital analbuminemia; CD, cluster of differentiation; IgG, immunoglobulin G, PLE, protein-losing enteropathy; RTE, recent thymic emigrant; Tem, effector memory T cells.

For contextual comparison, immunological data from patients with CAA were analyzed alongside age-matched control groups, including children with PLE, CHAPLE disease, and AT. Patients with CAA had higher serum IgG levels than patients with PLE, CHAPLE disease, and AT (*p* = 0.004, *p* = 0.005, and *p* = 0.001, respectively) (Figure 4A). Absolute lymphocyte counts were also higher in CAA compared with PLE and AT (*p* = 0.003 and *p* = 0.004, respectively) (Figure 4B). Total CD4⁺ T-cell counts were higher in CAA than in PLE and AT (both *p* = 0.003) (Figure 4C). Naive CD4⁺ T-cell counts were preserved in CAA and were higher than those observed in PLE and AT comparators (*p* = 0.03 and *p* < 0.001, respectively) (Figure 4D). Recent thymic emigrant frequencies were higher in CAA than in AT (*p* = 0.03), consistent with normal TREC values in the tested patients (Figure 4E). CD4⁺ effector memory T-cell frequencies were higher in CAA than in PLE (*p* = 0.04) (Figure 4F).

**Figure 5:**
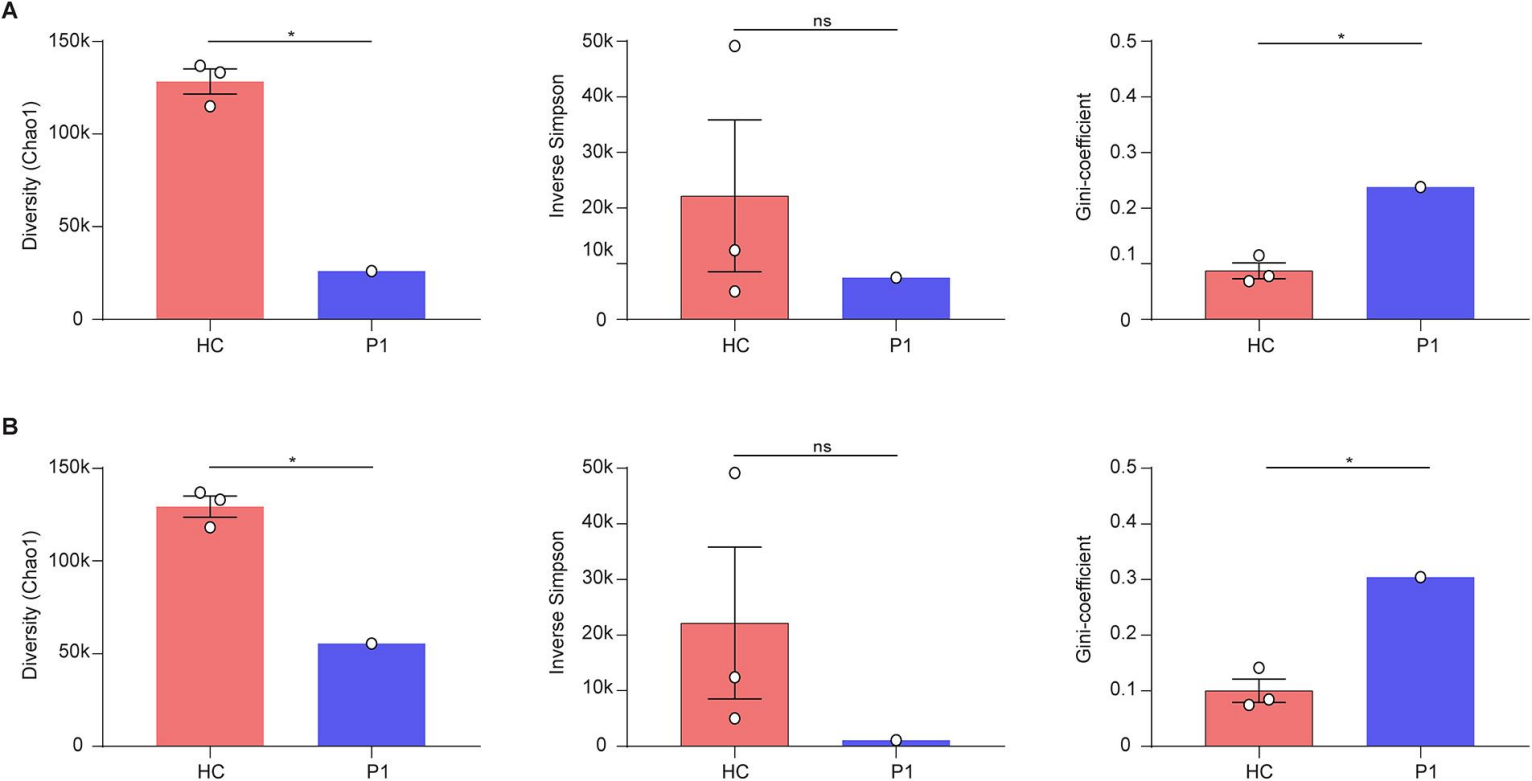
T-cell receptor *β* profile of patient P1. **(A and B)** Diversity and evenness analyses of T-cell repertoire. Diversity estimations were calculated using the Chao1 and Inverse Simpson indices. Inequality analysis was performed using the Gini coefficient measurement. Chao1, Inverse Simpson, and Gini indices are presented in **A** and **B**, respectively.

High-throughput T-cell receptor beta repertoire sequencing was performed in one patient. In this patient, CD4⁺ and CD8⁺ T-cell receptor diversity appeared to be restricted compared with that of healthy controls. The Chao1 diversity index was lower in both CD4⁺ and CD8⁺ T-cell CDR3 sequences, and clone distribution showed greater inequality, as reflected by a higher Gini coefficient (Figures 5A,B). Because this analysis was performed in a single patient, these findings should be interpreted as preliminary and not representative of the entire cohort.

## Discussion

CAA is traditionally considered a rare biochemical condition in which compensatory increases in non-albumin plasma proteins partially mitigate the absence of albumin. Here, by integrating genetic diagnosis with longitudinal clinical follow-up and targeted coagulation and immunological profiling in five pediatric patients, we provide an expanded view of the pediatric CAA phenotype and highlight clinically relevant vascular and immune features that warrant systematic attention in routine care.

From a clinical perspective, our cohort clearly demonstrates that CAA in childhood is far from silent. While adult patients often remain paucisymptomatic due to compensatory increases in non-albumin proteins, recent systematic reviews and case series have highlighted that pediatric patients frequently present with generalized edema, recurrent infections, dyslipidemia, and antenatal complications, such as polyhydramnios and hydrops fetalis (2, 4, 7). Consistent with these findings, all of these manifestations were evident in our cohort. Interestingly, all of our patients experienced GI symptoms such as diarrhea and abdominal pain, resulting in a misdiagnosis of PLE, which was an unusual finding given that GI symptoms have been infrequently reported in the literature. The occurrence of these symptoms is particularly significant and likely reflects intestinal wall edema due to altered oncotic pressure, a plausible mechanism (4). Notably, one patient presenting with hypocalcemic myoclonus has been sporadically described in the literature (2, 5, 20) linked to defective albumin-dependent calcium transport, highlighting the potential for under-recognition of non-classical features in pediatrics. Finally, the uniform presence of dyslipidemia across our patients aligns with prior studies showing compensatory shifts toward *β*- and *γ*-globulin fractions and broad lipid metabolic alterations, further reinforcing the concept of systemic metabolic reprogramming in the absence of albumin (21). Taken together, these data support an emerging paradigm that pediatric CAA is a symptomatic, multisystem disorder with clinically significant morbidity rather than a benign biochemical trait. Our findings, together with the existing literature, underscore the importance of molecular genetic testing in all children with unexplained, persistent hypoalbuminemia, particularly in consanguineous populations (2, 5). Our patients continued to require regular albumin infusions to manage edema and other symptoms, consistent with previous reports (2). While symptomatic benefit is clear, infusions do not prevent GI morbidity, dyslipidemia, infections, or TE. This underscores the need for individualized care protocols that extend beyond replacement therapy to include vascular risk surveillance and infection prevention.

Genetically, we expand the *ALB* mutational spectrum by identifying a novel homozygous nonsense (stop-gain) insertion in addition to confirming a recurrent splice-site mutation. More than 27 distinct loss-of-function *ALB* variants have been reported to date, including frameshift, nonsense, splice-site, and start-codon variants, most of which are family-specific (2). Despite this allelic heterogeneity, the core biochemical phenotype was consistent across our patients, with severe hypoalbuminemia and compensatory changes in non-albumin plasma protein fractions. However, this compensation seems incomplete, as systematic reviews show affected individuals often have plasma protein levels below the reference range. These reviews indicate that plasma protein upregulation is only partial and cannot replace albumin's roles in transport, buffering, and antioxidant defense (5, 21). In contrast to the relatively consistent biochemical phenotype, clinical expression varied substantially across patients. Notably, variability was observed even among siblings carrying the same *ALB* variant, suggesting that the causative genotype alone may not fully determine disease severity. Developmental stage, age at presentation, intercurrent illness, treatment exposure, and additional genetic or environmental modifiers may all contribute to phenotypic variability. This interpretation is consistent with the broader CAA literature, in which some individuals are diagnosed incidentally in adulthood with minimal symptoms, whereas others, particularly in infancy or childhood, experience severe fluid retention and clinically significant complications (2). Given the small cohort size, shared consanguineous background, and limited allelic diversity, robust genotype–phenotype correlations cannot be established in the present study. Larger multicenter cohorts with standardized clinical and molecular annotation are needed to determine whether specific *ALB* variant classes, residual albumin production, or modifier factors contribute to phenotypic variability in CAA.

One of the most notable observations in our cohort was the presence of coagulation abnormalities suggestive of a prothrombotic tendency. Although thrombotic complications have previously been reported in CAA, available data are largely limited to sporadic venous and arterial events in young adults, and systematic pediatric coagulation data remain scarce (9, 12, 20). In our cohort, all patients had elevated fibrinogen and D-dimer levels, and 4/5 patients showed increased FXI activity; one child experienced a life-threatening cerebral thrombosis. These findings suggest that an altered hemostatic profile may occur in pediatric CAA, although the small sample size and the presence of only one clinically documented TE event preclude firm conclusions regarding thrombotic risk. The elevation of FXI activity is particularly noteworthy, as this finding has not, to our knowledge, been systematically described in CAA. Elevated FXI activity has been associated with increased risk of venous TE in population-based studies (22–24). In parallel, the concurrent increases in protein C and protein S may reflect a compensatory anticoagulant response to coagulation activation rather than simple unidirectional hypercoagulability. Comparison with PLE showed differences in selected coagulation parameters, particularly FXI and natural anticoagulant activities. This raises the possibility that the hemostatic profile observed in CAA may not be explained by hypoalbuminemia alone. These observations are also biologically plausible in light of previous data linking low serum albumin levels with increased fibrinogen and FVIII levels and with venous TE risk (8). Moreover, experimental evidence suggests that albumin exerts antioxidant and antiplatelet effects, and its absence may promote platelet activation and thrombin generation (25). However, these mechanisms were not directly tested in the present study. Therefore, the coagulation findings should be interpreted cautiously, given the very small cohort size, cross-sectional design, potential inter-cohort heterogeneity, individualized albumin replacement exposure, and absence of functional coagulation or platelet assays.

Our immunological findings further broaden the disease spectrum. Unlike PLE or CHAPLE, where Ig wasting and lymphopenia dominate, the patients with CAA exhibited preserved or elevated IgG levels consistent with compensatory upregulation of non-albumin plasma protein fractions, including *γ*-globulins, as previously reported in clinical series and analbuminemic animal models (1, 2, 5). Vaccine responses were preserved for hepatitis B and rubella; however, the absence of responses to mumps and pneumococcal antigens in two patients indicates that antigen-specific humoral protection may be variable and should be assessed individually. Despite preserved major immune-cell compartments, more subtle immunological abnormalities were observed. KREC values, available in two patients, were reduced, and CSM B cells were decreased in the majority of the patients, suggesting a possible disturbance in B-cell generation or maturation. TREC values were within the expected range in the tested patients, and RTE frequencies were only mildly reduced or near the lower end of the reference range in some cases. Moreover, RTE frequencies were clearly higher in CAA than in immunodeficiency comparator groups, particularly CHAPLE disease and AT, supporting the interpretation that thymic output remains largely intact in CAA, in contrast to the marked thymic failure observed in combined immunodeficiencies. In the CD4⁺ T-cell memory compartment, EM cells expanded while CM cells decreased, indicating a shift toward an antigen-experienced phenotype. This pattern is consistent with the biology of chronic or repeated antigen exposure, in which continued antigenic stimulation promotes differentiation toward EM at the expense of the CM pool (26). Taken together, these findings suggest that the infectious burden in pediatric CAA may reflect immune dysregulation rather than overt combined immunodeficiency. Intact innate and adaptive immune responses depend on albumin; its absence or severe deficiency may affect interactions with bioactive lipid mediators, antimicrobial defense mechanisms, and endothelial integrity, providing a biologically plausible link between congenital albumin deficiency and increased infectious susceptibility (27). We hypothesize that severe congenital albumin deficiency may contribute indirectly to immune activation through altered oncotic balance, mucosal and tissue edema, recurrent respiratory and gastrointestinal inflammation, altered mediator transport, reduced antioxidant buffering, and endothelial instability. Repeated antigenic stimulation during infections or mucosal inflammation may contribute to EM CD4⁺ T-cell expansion, whereas reduced KREC values and CSM B-cell abnormalities may reflect altered B-cell generation, maturation, or survival under chronic inflammatory or metabolic stress. These interpretations remain hypothesis-generating, as cytokine profiling, oxidative stress measurements, endothelial assays, B-cell functional assays, and longitudinal pre- and postalbumin immune profiling were not performed. Clinically, the current findings do not support routine Ig replacement therapy in the absence of documented antibody failure, but do support individualized monitoring of vaccine responses, infection history, and immune parameters as part of systematic follow-up in children with CAA.

Albumin replacement therapy should be considered a major potential confounding factor in the interpretation of immune and coagulation findings. Beyond its oncotic effect, albumin has antioxidant, anti-inflammatory, transport, and immunomodulatory properties, and it may influence endothelial integrity, microcirculatory function, platelet activity, and coagulation dynamics. Previous studies have shown that albumin can modulate systemic inflammation and cytokine profiles, preserve endothelial glycocalyx integrity, and exert concentration-dependent effects on platelet aggregation and global coagulation assays (28–30).

Thus, the immune and coagulation abnormalities observed in our cohort cannot be assumed to reflect the untreated natural course of CAA. While blood samples were ideally collected during stable clinical periods and, when possible, several days post-infusion, the exposure to albumin was not completely standardized.

This study has several limitations. First, the cohort consisted of only five pediatric patients with CAA, reflecting the extreme rarity of the disease. This very small sample size limits statistical power, prevents robust subgroup analyses, and restricts the generalizability of the findings. Therefore, the results should be interpreted as descriptive and hypothesis-generating rather than definitive. Second, although control groups were age-matched and sampled during clinically stable periods, they were not matched for disease mechanism, inflammatory burden, nutritional status, disease duration, or treatment exposure. The absence of longitudinal follow-up in comparator groups further limited disease-specific interpretation. Third, conclusions regarding hypercoagulability should be interpreted cautiously. Coagulation abnormalities and one TE were observed; however, the small cohort size, cross-sectional design, variable albumin exposure, and lack of functional coagulation assays preclude firm conclusions regarding thrombotic risk or causality. Fourth, no functional experiments were performed to validate the proposed mechanisms. Cytokine stimulation assays, platelet function studies, endothelial assays, oxidative stress measurements, *in vitro* albumin-replacement experiments, and animal-model validation were not available. In addition, some advanced immunological analyses, including TCR repertoire assessment, were performed in only a subset of patients or in a single patient, limiting these findings to an exploratory status. Finally, all patients came from consanguineous families and were evaluated in tertiary referral centers, which may have introduced genetic, environmental, and referral-related biases. Although genotype–phenotype observations are clinically relevant, this cohort is too small to establish reliable correlations. Overall, this study provides detailed multisystem phenotyping of pediatric CAA, but the findings should be interpreted within the constraints of a very small, treated, cross-sectional cohort. Larger multicenter studies with standardized documentation of albumin treatment, precisely timed sampling, matched control groups, longitudinal follow-up, and functional validation are required.

## Conclusions

In this pediatric cohort, CAA was associated with persistent severe hypoalbuminemia and a broad clinical phenotype including GI morbidity, dyslipidemia, recurrent infections, and abnormalities in coagulation and immune parameters. These findings support the concept that CAA may represent a multisystem disorder rather than an isolated biochemical abnormality. Early molecular diagnosis may help reduce unnecessary invasive investigations, guide supportive treatment, and facilitate targeted monitoring for infectious and thrombotic complications.

This study provides clinically relevant phenotypic data on pediatric CAA and highlights the need for larger multicenter studies with longitudinal follow-up and functional validation to better define the systemic effects of albumin deficiency and its potential role in vascular and immune homeostasis.

## Data availability statement

The data generated during the study are included in this published article and its Supplementary File.

## Ethics statement

This study involving humans was approved by the Ethics Committees of Marmara University and Sidra Medicine. This study was conducted in accordance with the local legislation and institutional requirements. Written informed consent for participation in this study was provided by the participants' legal guardians/next of kin. Written informed consent was obtained from the individual(s), and minor(s)' legal guardian/next of kin, for the publication of any potentially identifiable images or data included in this article.
