# Supplementary material for: Genomic diagnosis and multisystem phenotyping in pediatric congenital analbuminemia: clinical, coagulation, and immune signatures
Source: Front Pediatr. 2026 Jun 2;14:1810945. doi: 10.3389/fped.2026.1810945 (PMC13269094; doi:10.3389/fped.2026.1810945)
Supplement: Supplementary file 1 [file Datasheet1.pdf]

Supplementary Material

Supplementary Methods

S1. Genomic Analysis and Interpretation

Genomic DNA was extracted from peripheral blood using the QIAamp DNA Blood Mini Kit (Qiagen). Trio whole-genome sequencing (WGS) was performed at Sidra Medicine (Qatar) on the Illumina HiSeq X platform, targeting an average genome-wide coverage of ~30×. Sequencing reads were quality-filtered and aligned to the human reference genome (GRCh37/hg19) using Burrows–Wheeler Aligner (BWA-MEM) with default parameters. Single-nucleotide variants (SNVs) and small insertions/deletions (INDELs) were called using the Genome Analysis Toolkit (GATK) pipeline and annotated by the Sidra Bioinformatics Core using standard annotation tools.

Variant prioritization was performed within trios using inheritance- and phenotype-driven filtering, with emphasis on autosomal recessive models when supported by family structure. Variants were filtered using standard quality metrics, including minimum read depth (RD > 20) and genotype quality (GQ > 15). Variants were further filtered by population frequency (minor allele frequency < 0.01) in reference databases, including gnomAD and the 1000 Genomes Project. Variant allele fraction (VAF) was reviewed to reduce low-level artefacts; variants with VAF > 0.25 were retained for downstream assessment, with final prioritization guided by expected zygosity patterns within trios and manual review when needed. Synonymous variants were deprioritized unless predicted to affect splicing.

Predicted functional impact was assessed using in-silico tools (SIFT, PolyPhen-2, and MutationTaster), conservation metrics (e.g., GERP), and aggregated scores such as CADD. Computational metrics were used as supportive evidence and for ranking rather than as sole decision thresholds. Final variant classification was performed according to the ACMG/AMP guidelines, integrating population data, predicted molecular consequence (including splice-site impact when applicable), segregation within trios, computational evidence, and phenotype consistency. The in-house Variant Analysis with Multiple Pathogenicity Predictors (VAMPP) score was used only as an auxiliary ranking metric and did not replace ACMG/AMP classification.

Candidate ALB variants were confirmed by Sanger sequencing. Briefly, genomic DNA was amplified by polymerase chain reaction, and amplicons were sequenced using the BigDye Terminator v1.1 Cycle Sequencing Kit (Applied Biosystems; Life Technologies, Darmstadt, Germany) on an Applied Biosystems 3130 Genetic Analyzer.

S2. Antibodies and Flow Cytometry

Lymphocyte immunophenotyping was performed on peripheral whole-blood samples using multiparameter flow cytometry. The following monoclonal antibodies (mAbs) were used: FITC-CD3 (UCHT1, 1:50, Beckman Coulter, France), APC-CD3 (33-

2A3, 1:50, Immunostep, Spain), APC-A700-CD4 (13B8.2, 1:50, Beckman Coulter), FITC-CD4 (HP2/6, 1:50, Immunostep), APC-CD4 (HP2/6, 1:50, Immunostep), Krome Orange-CD45 (J33, 1:50, Beckman Coulter), APC-A750-CD45RA (2H4DH11LDB9, 1:50, Beckman Coulter), PE-CD197/CCR7 (G043H7, 1:50, Beckman Coulter), PC7-CD8 (SFCI21Thy2D3, 1:50, Beckman Coulter), APC-A700-CD14 (RMO52, 1:50, Beckman Coulter), PE-CD16 (3G8, 1:50, Beckman Coulter), PC5.5-CD56 (N901, 1:50, Beckman Coulter), APC-A750-CD19 (J3-119, 1:50, Beckman Coulter), PB-CD20 (B9E9, 1:50, Beckman Coulter), PB-CD21 (BL13, 1:50, Beckman Coulter), PB-CD31 (5.6E, 1:50, Beckman Coulter), PC5.5-CD38 (LS198-4-3, 1:50, Beckman Coulter), ECD-CD45RO (UCHL1, 1:50, Beckman Coulter), FITC-IgD (IA6-2, 1:50, Beckman Coulter), PE-PD-1 (PD1.3, 1:50, Beckman Coulter), APC-CXCR5 (J252D4, 1:50, Beckman Coulter), PC5.5-CD25 (B1.49.9, 1:40, Beckman Coulter), PE/Cy7-CD25 (2A3, 1:50, BD Biosciences, CA, USA), Alexa Fluor 647-FOXP3 (259D, 1:20, BD Biosciences), PE-ICOS/CD278 (C398.4A, 1:100, BioLegend, CA, USA), Alexa Fluor 488-NF-κB p65 (pS529; K10-895.12.50, 1:20, BD Biosciences), and CellTrace™ CFSE (C34554, Invitrogen, MA, USA).

For lymphocyte subset analysis, 100 µL of whole blood was incubated with antibodies against surface markers at room temperature in the dark for 20 min. Red blood cells were subsequently lysed according to the manufacturer's instructions, and samples were washed prior to acquisition. Data were acquired on a Navios EX flow cytometer (Beckman Coulter) and analyzed using Kaluza Analysis Software (v2.1). Lymphocytes were identified by forward/side scatter characteristics and CD45 expression, with sequential gating to define major lineages and maturation subsets. Instrument performance was monitored using routine daily quality-control procedures in accordance with institutional protocols.

T-cell subsets were defined using CD45RA and CCR7 as follows: CD4$^+$ naïve (CD4$^+$CD45RA$^+$CCR7$^+$), central memory (CD4$^+$CD45RA$^-$CCR7$^+$), effector memory (CD4$^+$CD45RA$^-$CCR7$^-$), and TEMRA (CD4$^+$CD45RA$^+$CCR7$^-$). Analogous subsets were defined for CD8$^+$ T cells. B-cell subsets were defined as naïve mature B cells (CD19$^+$CD27$^-$IgD$^+$), non-class-switched memory B cells (CD19$^+$CD27$^+$IgD$^+$), class-switched memory B cells (CD19$^+$CD27$^+$IgD$^-$), and CD21^lowCD38^low B cells. Where applicable, additional functional/activation markers (e.g., PD-1, ICOS, CXCR5, CD25, FOXP3, and NF-κB p65 phosphorylation) were assessed using standard gating strategies within parent populations.Subset proportions were compared with age-specific normative data (1), healthy controls (HC), and disease controls where relevant, including ataxia-telangiectasia (AT) and CHAPLE disease, to provide a clinical context for interpretation.

S3. TREC and KREC Quantification

T-cell receptor excision circles (TRECs) and kappa-deleting recombination excision circles (KRECs) were quantified by quantitative real-time PCR (qPCR) using institutional protocols. Copy numbers were determined using standard curve-based quantification and interpreted relative to age-matched reference values. Where available, results were evaluated alongside contemporaneous lymphocyte subset counts to support integrated interpretation of thymic output and B-cell neogenesis.

S4. T-cell Receptor Immune Repertoire Analysis (TRA/TRB Profiling)

Peripheral blood mononuclear cells (PBMCs) were isolated from peripheral blood by density-gradient centrifugation using standard institutional protocols. $CD4^+$ and $CD8^+$ T cells were enriched from PBMCs using the MojoSort Human CD4 and CD8 T Cell Isolation Kits (BioLegend). Post-isolation purity was confirmed to be >95% by flow cytometry. Total RNA was extracted from isolated T-cell fractions using the NucleoSpin RNA Plus Kit (Macherey-Nagel). T-cell receptor alpha (TRA) and beta (TRB) libraries were prepared from 50 ng RNA using the SMARTer Human T-cell Receptor (TCR) α/β Profiling Kit (Takara) according to the manufacturer's instructions. Library quality was assessed by amplicon size distribution using an Agilent Bioanalyzer 2100 with the DNA 1000 Kit. Sequencing was performed on an Illumina MiSeq platform. Raw immune repertoire data were processed using Cogent NGS Immune Profiler Software (Takara) to generate clonotype tables and V/J gene assignments. Downstream repertoire analyses were performed in R (RStudio) using the Immunarch package. Diversity and clonality were summarized using standard metrics, including Chao1 (richness), the inverse Simpson index (diversity), and the Gini coefficient (inequality/clonality). Where required to account for sequencing depth, samples were normalized using a consistent read-based approach (e.g., rarefaction/downsampling) prior to cross-sample comparisons. Pathology-associated TRB sequences were queried against curated public databases (McPAS-TCR and TCRdb) using CDR3 amino acid sequence matching, with V/J assignment when available. Given the limited sample size and specimen availability, repertoire analyses were interpreted as exploratory (2–3).

Supplementary Patients' Descriptions

S6. Patient 1

Patient 1 is a 17-year-old female born to consanguineous parents, with an unremarkable antenatal and perinatal history. She became symptomatic in early infancy (approximately 2 months of age) with persistent watery diarrhea and progressive generalized edema, initially most prominent in the periorbital region and lower extremities. Early laboratory investigations revealed severe hypoalbuminemia, while renal function tests, urinalysis, and liver function parameters were repeatedly

normal. Given the coexistence of gastrointestinal symptoms and hypoalbuminemia, she was followed for years with a working diagnosis of protein-losing enteropathy (PLE). Throughout infancy and early childhood, the patient experienced recurrent gastrointestinal complaints, including chronic diarrhea and abdominal distension, with fluctuating severity. Endoscopic evaluation performed during childhood demonstrated intestinal lymphangiectasia, although this finding was not consistently reproducible in subsequent examinations. Despite extensive gastroenterological follow-up, no definitive secondary cause of hypoalbuminemia was identified.

Persistent hypoalbuminemia necessitated regular intravenous albumin infusions from early childhood, which provided transient clinical improvement with partial resolution of edema; however, symptoms recurred, and complete biochemical normalization was not achieved. Dietary interventions, including high-protein intake and medium-chain triglyceride supplementation, provided limited and short-lived benefit. In parallel with gastrointestinal morbidity, she had recurrent respiratory tract infections involving both the upper and lower airways. Several episodes required hospitalization, and at least one episode was complicated by sepsis. Cultures from severe infections identified Haemophilus influenzae in respiratory samples and Staphylococcus aureus in blood cultures. Infectious episodes were associated with clinical deterioration, worsening edema, and increased albumin requirements.

At 15 years of age, during a period of pronounced hypoalbuminemia (serum albumin 1.8 g/dL), the patient developed acute neurological symptoms. Neuroimaging revealed an extensive cerebrovascular thromboembolic event with basilar artery occlusion and multiple cerebral infarctions. She required intensive care admission, endotracheal intubation,

emergency thrombectomy, and systemic anticoagulation. The clinical course was complicated by cerebral edema, requiring external ventricular drainage and decompressive craniectomy. Prior to this event, the patient had normal neuromotor development and age-appropriate cognitive function. Following the thromboembolic event, she developed severe neurological sequelae, including left-sided hemiplegia with markedly reduced muscle strength, facial paralysis, impaired gaze, dysarthria, and cognitive deficits. She remains wheelchair-dependent and requires ongoing physiotherapy, long-term anticoagulation, and continued albumin replacement therapy. During adolescence, she developed lower-extremity lipodystrophy.

Trio WGS identified a homozygous canonical splice-site variant in ALB (c.1428+2T>C), predicted to abolish normal albumin production. The variant was confirmed by Sanger sequencing, establishing the diagnosis of congenital analbuminemia.

At last follow-up, she continues to require regular intravenous albumin replacement therapy to control edema and maintain functional well-being and remains on long-term anticoagulant therapy for secondary prevention.

S7. Patient 2

Patient 2 is a 15-year-old female born to consanguineous parents. Antenatal history was notable for polyhydramnios. She became symptomatic at 54 months of age with episodic abdominal pain and generalized edema. During early childhood, she also developed intermittent myoclonic movements, which were attributed to hypocalcemia in the context of profound hypoalbuminemia. Renal and hepatic evaluations were repeatedly normal.

Given persistent hypoalbuminemia with intermittent gastrointestinal symptoms, she was followed with a working diagnosis of PLE. Endoscopic evaluation and histopathology were unremarkable, and fecal studies did not provide consistent evidence of protein loss. She required intermittent intravenous albumin supplementation with transient symptomatic improvement, particularly for edema. She did not experience thromboembolic complications or severe infections. Trio WGS identified a homozygous ALB frameshift variant (c.204_205ins; p.(Glu69Ter)), establishing the diagnosis of congenital analbuminemia.

At last follow-up, she received episodic albumin replacement during periods of clinical worsening and was not receiving long-term anticoagulation or antibiotic prophylaxis.

S8. Patient 3.1

P3.1 17-year-old female from the same kindred as P3.2 and P3.3. Antenatal history was remarkable for hydrops fetalis. She became symptomatic in infancy (approximately 12 months of age) with generalized edema and chronic diarrhea. Persistent hypoalbuminemia was documented early in life, and renal and hepatic causes were excluded. She experienced recurrent lower respiratory tract infections

during childhood, including pneumonia, associated with clinical deterioration and increased albumin requirements. Intestinal biopsies demonstrated lymphoplasmacytic infiltration of the lamina propria. Long-term albumin replacement therapy was required to control edema and gastrointestinal symptoms. During adolescence, she developed lower-extremity lipodystrophy. No thromboembolic events have been documented to date.

Genetic analysis revealed homozygosity for the ALB frameshift variant (c.204_205ins; p.(Glu69Ter)), confirming congenital analbuminemia. At last follow-up, she continues to require regular albumin supplementation and remains under multidisciplinary follow-up.

S9. Patient 3.2

Patient 3.2 is an 11-year-old female sibling of P3.1 who became symptomatic at 60 months of age with episodic abdominal pain and intermittent peripheral edema. She experienced recurrent upper respiratory tract infections but did not develop severe or invasive infections. Gastrointestinal evaluation, including endoscopy and histopathology, was unremarkable. She requires periodic intravenous albumin supplementation to control edema. No thromboembolic events have been documented. Genetic testing identified the same homozygous ALB frameshift variant (c.204_205ins; p.(Glu69Ter)) as in her affected siblings. At present, she is not receiving anticoagulant therapy or antibiotic prophylaxis and continues to be monitored for infectious and thrombotic complications.

S10. Patient 3.3

Patient 3.3 is a 5-year-old female from a consanguineous family and the youngest affected sibling. She presented at 24 months of age with generalized edema and chronic watery diarrhea. Laboratory evaluation demonstrated persistent hypoalbuminemia in the absence of renal or hepatic protein loss. Endoscopy revealed ectatic intestinal lymphatic vessels, leading to an initial diagnosis of intestinal lymphangiectasia/PLE. Despite nutritional interventions and albumin replacement, serum albumin levels remained markedly low.

She has not experienced recurrent or severe infections. Trio WGS revealed homozygosity for the ALB frameshift variant (c.204_205ins; p.(Glu69Ter)), confirming congenital analbuminemia. She remains under close surveillance and receives albumin replacement during periods of symptomatic edema.

Supplementary Table 1: Detailed immunological findings of congenital analbunemia patients.

**Subject**	**P1.1**	**P2**	**P3.1**	**P3.2**	**P3.3**
**Absolute lymphocyte count (/mm^3)**	1760	1730	4410	3600	3400
**Absolute neutrophil count (/mm^3)**	4010	3200	2980	2200	5100
**Immunoglobulins**					
IgA (mg/dL)	289 (108- 477)	230 (96-465)	78 (71-235)	181 (69-387)	199 (108- 477)
IgG (mg/dL)	**2400 (↑)** (876- 2197)	**1980 (↑)** (987-1958)	869 (640-2810)	1276 (764-2134)	1475 (876-2197)
IgM (mg/dL)	310 (75-448)	280 (83-282)	183 (44-244)	156 (78-383)	174 (75-448)
IgE (IU/ml)	**250 (↑)**	**126 (↑)**	**348 (↑)**	**100 (↑)**	**89 (↑)**
**Vaccine Responses**					
Anti-Hbs	positive	positive	positive	positive	positive
Anti-Mumps	negative	negative	positive	positive	positive
Anti-Rubeola	positive	positive	positive	positive	positive
Anti-Pneumococcus	negative	negative	positive	positive	positive
**TREC median (min-max)**	**998 (↓)** 12600 (720-36200)	**1150 (↓)** 12600 (720-36200)	NA	NA	NA
**KREC median (min-max)**	**1320 (↓)** 11335 (1720-61000)	**914 (↓)** 11335 (1720-61000)	NA	NA	NA
**Lymphocyte subsets**					
CD3+ T cells, (%)	63 (59-88)	77 (59-88)	71 (58-83)	75 (56-89)	82 (59-88)
CD3+CD4+ T cells, (%)	39 (28-48)	46 (28-48)	41 (25-55)	46 (25-51)	45 (28-48)
CD3+CD8+ T cells, (%)	18 (18-43)	20 (18-43)	24 (14-39)	26 (18-43)	23 (8-43)
CD19+ B cells, (%)	**26 (↑)** (5-21)	14 (5-21)	21 (10-31)	17 (7-23)	12 (5-21)
CD16+56+ NK cells, (%)	10 (5-35)	6 (5-35)	5 (3-30)	6 (4-29)	6 (5-35)
CD19+CD27-IgD+ B cells, (%)	65 (46-92)	70 (46-92)	88 (55-95)	77 (55-90)	77 (46-92)
CD19+CD27+IgD+ B cells, (%)	11 (5-28)	26 (5-28)	**3 (↓)** (6-23)	18 (6-28)	9 (5-28)
CD19+CD27+IgD- B cells, (%)	12 (6-35)	**2 (↓)** (6-35)	**1 (↓)** (3-32)	**3 (↓)** (7-31)	**3 (↓)** (6-35)
CD21low CD38low activated B, (%)	4 (1-15)	3 (1-15)	1 (1-11)	6 (2-13)	5 (1-15)
CD3+ TCRα/β cells, (%)	86 (79-99)	88 (79-99)	88 (79-100)	89 (73-100)	90 (79-99)
CD3+ TCRγ/δ cells, (%)	12 (4-25)	7 (4-25)	7 (3-27)	8 (4-29)	5 (4-25)
CD4+ CD45RA+ CD31+ T cells, (%)	**22 (↓)** (28-61)	37 (28-61)	59 (49-78)	**34 (↓)** (37-73)	**27 (↓)** (28-61)
CD4+ CD45RA+ CCR7+ T cells, (%)	32 (31-70)	42 (31-70)	65 (49-90)	47 (40-79)	42 (31-70)
CD4 + CD45RA- CCR7+ T cells, (%)	**10 (↓)** (26-53)	17 (26-53)	**9 (↓)** (13-44)	**14 (↓)** (17-53)	**12 (↓)** (26-53)
CD4+ CD45RA- CCR7- T cells, (%)	**56 (↑)** (4-23)	**35 (↑)** (4-23)	**13 (↑)** (1-10)	**36 (↑)** (2-14)	23 (4-23)
CD4+ CD45RA+ CCR7- T cells, (%)	2 (1-21)	7 (1-21)	13 (1-58)	4 (1-43)	13 (1-21)
CD8+ CD45RA+ CCR7+ T cells, (%)	**15 (↓)** (17-72)	33 (17-72)	56 (21-100)	29 (22-67)	21 (17-72)
CD8+ CD45RA- CCR7+ T cells, (%)	3 (2-14)	8 (2-14)	5 (1-9)	8 (2-10)	3 (2-14)
CD8+ CD45RA- CCR7- T cells, (%)	44 (10-45)	30 (10-45)	11 (5-33)	32 (7-41)	36 (10-45)
CD8+ CD45RA+ CCR7- T cells, (%)	36 (15-77)	29 (15-77)	28 (13-64)	31 (18-74)	67 (15-77)

CD: Cluster Differentiated, CCR7: Chemokine receptor 7, Ig: Immunoglobulin, KREC: Kappa-deleting Recombination Excision Circle, NA: Not available, NK: Natural Killer, TCR: T Cell Receptor, TREC: T-cell Receptor Excision Circle, Reference ranges (given in parentheses) are age-specific. ↑: Above age-matched reference range, ↓: Below age-matched reference range. Values outside the age-specific reference ranges are indicated in bold.

References

1) Besci, O.; Baser, D.; Ogulur, I.; et al. Reference values for T and B lymphocyte subpopulations in Turkish children and adults. Turk. J. Med. Sci. 2021, 51, 1814–1824.

2) Aba, U.; Maslak, I.C.; Ipsir, C.; et al. A novel homozygous germline mutation in transferrin receptor 1 (TfR1) leads to combined immunodeficiency and provides new insights into the iron–immunity axis. J. Clin. Immunol. 2024, 44, 55.

3) Fang, M.; Su, Z.; Abolhassani, H.; et al. T cell repertoire abnormality in immunodeficiency patients with DNA repair and methylation defects. J. Clin. Immunol. 2022, 42, 375–393.
